# Research progress on pathogenesis of skin pigmentation in chronic liver disease

**DOI:** 10.17305/bb.2024.11085

**Published:** 2024-12-10

**Authors:** Tianqi Liu, Tianyu Xi, Xiaoqin Dong, Dong Xu

**Affiliations:** 1 The Second Clinical Medical College, Tongji Medical College, Huazhong University of Science and Technology, Wuhan, China; 2 Department and Institute of Infectious Disease, Tongji Hospital, Tongji Medical College and State Key Laboratory for Diagnosis and Treatment of Severe Zoonotic Infectious Disease, Huazhong University of Science and Technology, Wuhan, China

**Keywords:** Skin pigmentation, chronic liver disease, CLD, liver cirrhosis, pathogenesis

## Abstract

Chronic liver disease (CLD) is a significant global health concern that leads to increased morbidity and mortality, and is associated with skin pigmentation changes. Excessive facial pigmentation is a common characteristic of patients with CLD, although the exact mechanism underlying this phenomenon remains unclear. Melanin, which consists of eumelanin and pheomelanin, is synthesized in melanocytes. Its production is influenced by cysteine levels and is regulated by key enzymes, such as tyrosinase (TYR), tyrosinase-related protein 1 (TYRP1), and tyrosinase-related protein 2 (TYRP2). The transport of melanosomes within melanocytes relies primarily on the coordinated action of F-actin and microtubules. However, the mechanism of melanin transfer from melanocytes to surrounding dendritic cells requires further investigation. Several factors contribute to liver fibrosis, including oxidative stress and inflammatory cytokines. This article discusses the factors that are elevated in the serum of patients with chronic liver disease, which may increase melanin deposition. It also introduces the signaling pathways related to melanin synthesis, providing indirect evidence for the pathological mechanisms underlying increased melanin synthesis in CLD. Additionally, the article points out that pigmentation may serve as an important indicator of liver disease deterioration and suggests the formation of a scoring system that combines related factors to enhance the predictive accuracy. In terms of treatment, antioxidants and anti-inflammatory drugs, such as silymarin and vitamin E, may improve CLD and reduce skin pigmentation, but their specific effects still require further investigation. Future research should focus on validating the mechanisms linking pigmentation changes with CLD progression, and exploring therapeutic methods that can simultaneously improve liver function and skin pigmentation, ultimately aiming for better patient outcomes.

## Introduction

### Background

Chronic liver disease (CLD) is a significant global health threat and has become one of the leading causes of death in humans. The development of CLD is associated with various factors, the most common of which include alcohol abuse, obesity or metabolic disorders, autoimmune hepatitis, and viral hepatitis (HBV and HCV) [1]. Among these factors, non-alcoholic fatty liver disease (NAFLD) is the most prevalent, accounting for over 50% of all cases. The spectrum of NAFLD varies widely, ranging from simple steatosis to more severe forms, including progressive non-alcoholic steatohepatitis (NASH), which is characterized by inflammation and additional hepatocyte injury. This condition may further progress to cirrhosis, significantly increasing the risk of hepatocellular carcinoma (HCC) [2].

Liver fibrosis, as the progression of CLD, may influence the occurrence and development of facial pigmentation. Excessive facial pigmentation in CLD, commonly observed in clinical practice, is known as “liver disease face” or “hepatic face.” It is characterized by several distinct features, including a dull or dark complexion, periorbital pigmentation, dryness, rough skin texture, and poor elasticity. In some cases, individuals may also exhibit a “bronze-like” appearance [3].

### Knowledge gap and significance

However, despite the clinical prevalence of pigmentation in CLD, research on this phenomenon is limited and the exact underlying mechanisms remain unclear. This lack of understanding hinders clinicians’ ability to effectively use skin pigmentation as a diagnostic or monitoring tool in clinical practice.

**Figure 1.**
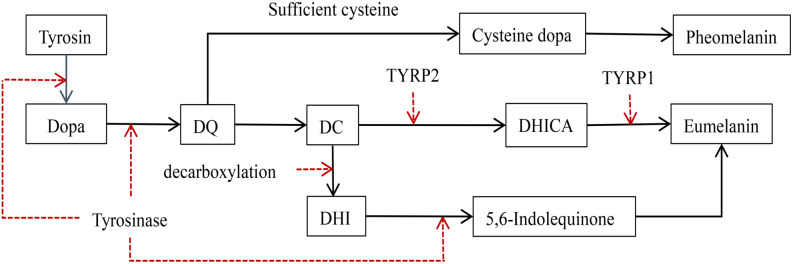
**The synthesis process of melanin.** Tyrosine is transported into melanosomes by TYRP1 and converted to L-DOPA by TYR. L-DOPA is oxidized to DQ. The synthesis ratio within a cell is influenced by the cysteine content. With sufficient glutathione and cysteine, DQ binds with glutathione to form 3-cysteinyl-DOPA and 5-cysteinyl-DOPA, which polymerize to produce pheomelanin. As cysteine levels decrease, DQ undergoes polymerization and oxidation to form DC. DC is converted to brownish DHICA by TYRP1 and TYRP2, or decarboxylates to form DHI. DHICA and DHI together form the second type of melanin. DQ: Dopaquinone; DC: Dopachrome; DHICA: Dihydroxyindole-2-carboxylic acid; DHI: 5,6-dihydroxyindole; TYRP1: Tyrosinase-related proteins 1; TYR: Tyrosinase; TYRP2: Tyrosinase-related proteins 2.

Case reports have confirmed that skin hyperpigmentation can occur during the deterioration of CLD and in acute-on-chronic liver failure (ACLF) [4–6]. Additionally, the presence of pigmentation may serve as a visual marker of changes in liver function, which could prompt timely intervention. This review aims to provide insight into the potential mechanisms of pigmentation in CLD, providing clues for predicting disease progression or the onset of acute liver failure, and suggesting potential methods to prevent pigmentation by targeting liver function. Ultimately, a better understanding of this phenomenon could lead to improved patient outcomes through early detection and management strategies.

### Objective of the review

This review explores the pathogenesis of hepatic facies, focusing on the role of liver fibrosis-related factors in melanin synthesis, transport, and signaling pathways. By examining these pathways, this review aims to uncover the complex interactions between liver disease and skin pigmentation. It combines pigment deposition with CLD, summarizing the factors that promote melanin synthesis in CLD, such as oxidative stress, inflammatory cytokines, and other proteins. Understanding these factors is essential to identify potential therapeutic targets. This review seeks to provide a comprehensive framework that can guide future research and clinical practices aimed at managing hepatic facies in patients with liver diseases.

## Overview of melanin synthesis and transport

### Melanin synthesis

Melanin, which is a key determinant of skin color, is comprised of eumelanin and pheomelanin. Melanin is formed and stored in melanosomes, which are located in the cytoplasm of the mature melanocytes. Melanocytes are predominantly found at the junction of the epidermis and dermis [7]. Melanin synthesis is a complex process that involves various factors. For instance, stem cell factor (SCF) plays a crucial role in the maturation of melanocytes by promoting the proliferation, differentiation, and migration of peripheral tissues, such as the skin and uvea [8].

The human melanin synthesis pathway, first proposed by Raper in 1926 and demonstrated by Manson in 1948, is known as the Raper–Manson pathway [9, 10]. Tyrosine is initially transported into melanosomes by tyrosinase-related protein 1 (TYRP1) and converted into L-3,4-dihydroxyphenylalanine (L-DOPA) by tyrosinase (TYR). L-DOPA is further oxidized to dopaquinone (DQ). Within a single cell, the synthesis ratio is primarily determined by cysteine content [11]. When sufficient glutathione and cysteine are available, DQ binds to glutathione and, with the participation of cysteine, forms 3-cysteinyl-DOPA and 5-cysteinyl-DOPA. These compounds undergo oxidative polymerization to produce pheomelanin, the first type of melanin. As cysteine levels decrease, DQ undergoes polymerization and oxidation reactions, generating dopachrome (DC). DC is converted by TYRP1 and tyrosinase-related protein 2 (TYRP2) to produce brownish dihydroxyindole-2-carboxylic acid (DHICA). Simultaneously, DC can decarboxylate TYR to form 5,6-dihydroxyindole (DHI). DHICA and DHI, after undergoing certain reaction steps, constitute the second type of melanin [12].

Therefore, TYR, TYRP-1, and TYRP-2 are critical enzymes in the process of melanin formation [8], and the synthesis rate of TYRP-1 positively correlates with the melanin synthesis rate (Figure 1) [13].

### Transport process of melanin

Melanosome transport occurs initially within melanocyte cells. During the onset of melanin synthesis, melanosomes containing fully synthesized melanin migrate from the perinuclear region to the dendrites of melanocytes under the coordinated action of F-actin and tubulin [8]. The motor proteins kinesin and dynein within tubulin coordinate with one another. Kinesin is responsible for long-distance movement toward the dendritic ends, while dynein drives melanosomes in the opposite direction [14, 15]. F-actin, which is enriched in dendritic ends, is connected to myosin-Va and myosin-VI. Myosin-Va drives melanosomes outward from melanocytes, whereas myosin-VI moves them in the opposite direction. Upon binding to F-actin, melanosomes are confined to the dendritic ends and undergo short-distance movements along the F-actin tracks [16, 17]. Ultimately, dendrites bind with 30–40 keratinocytes, transferring mature melanosomes into their cytoplasm [7].

However, the mechanism by which melanin is transferred from melanocytes to keratinocytes remains unclear. Proposed mechanisms include phagocytosis of melanocyte dendrites by keratinocytes, membrane fusion between cells, shedding of melanosome-rich microvesicles that are then engulfed by keratinocytes, and endocytosis of exposed melanin cores after exocytosis [18].

Therefore, it can be concluded that during the transport of melanosomes, kinesin facilitates the movement of melanosomes toward the dendritic ends, while myosin-Va is responsible for the outward movement of melanosomes at the dendritic tips. F-actin, connected to myosin-Va, confines melanosomes at the dendritic ends, ultimately facilitating their transfer from melanocytes to keratinocytes. Moreover, the specific mechanisms by which melanosomes are transferred from melanocytes to keratinocytes remain unclear.

## The relevant signaling pathways involved in melanin synthesis

Melanin biosynthesis involves multiple signaling pathways, primarily the melanocortin 1 receptor (MC1R)/α-melanocyte stimulating hormone (α-MSH) signaling pathway, phosphoinositide 3-kinase (PI3K)/Akt signaling pathway, Wnt/β-catenin signaling pathway, mitogen-activated protein kinase (MAPK) signaling pathway, and nitric oxide (NO) signaling pathway [19–22]. The transcription factor microphthalmia-associated transcription factor (MITF) serves as a key target in these pathways, and its activation leads to the upregulation of critical genes, such as *TYR*, *TYRP-1*, and *TYRP-2*, thereby promoting melanin production in melanocytes [23].

### MC1R/**α**-MSH signaling pathway

The cAMP/protein kinase A (PKA) signaling pathway is one of the most important signaling pathways. MC1R is a G protein-coupled receptor that is located on the surface of melanocytes. When α-MSH binds to MC1R, it activates adenylyl cyclase (AC), which increases intracellular cAMP levels [24]. This activates PKA, which phosphorylates and activates CREB-binding protein (CBP), leading to increased expression of *MITF* and regulation of melanin synthesis [25, 26]. In addition to α-MSH, adrenocorticotropic hormone (ACTH) can also act as an agonist of human MC1R, increasing cAMP levels and regulating melanin production [27, 28]. Therefore, MC1R is a major regulator of pigmentation in humans.

### PI3K/Akt signaling pathway

In addition to activating PKA activity, intracellular cAMP can upregulate *MITF* through the PI3K/Akt signaling pathway to regulate melanin production [29]. PI3K is activated when cAMP levels increase inside the cells. It generates two phospholipid products: 3,4-diphosphoinositide (PI-3,4-P2) and 3,4,5-triphosphoinositide (PI-3,4,5-P3). Subsequently, these products bind to Akt, leading to its phosphorylation and activation. Activated Akt inactivates glycogen synthase kinase 3 β (GSK3β). Reduced activity of GSK3β enhances the binding affinity between *MITF* and its box, further upregulating melanin production [30].

### Wnt/β-catenin signaling pathway

The Wnt signaling pathway plays a crucial role in embryonic development, cell proliferation, differentiation, and migration [31]. Wnts are secreted glycoproteins rich in cysteine that activate the Wnt signaling pathway upon binding to the Frizzled family seven-transmembrane receptor (Fzd2R) and low-density lipoprotein receptor-related protein 5/6 (LRP-5/6). This activation leads to the inactivation of GSK3β, preventing β-catenin from being phosphorylated by GSK3β, thereby reducing its ubiquitination and degradation and leading to β-catenin accumulation in the cytoplasm and translocation to the nucleus [32]. In the nucleus, β-catenin forms a complex with lymphoid enhancer factor/T-cell factor (LEF-TCF), which enhances the expression of *MITF* and stimulates melanin production [33, 34].

### MAPK signaling pathway

The MAPK signaling pathway also plays a significant role in melanin formation, involving MAPK family proteins, such as extracellular signal-regulated kinase 1/2 (ERK1/2), c-Jun N-terminal kinase (JNK), and p38. Binding of SCF to the cell surface c-Kit receptor activates Ras, which in turn activates B-Raf, initiating the MAPK signaling cascade. Activation of ERK leads to phosphorylation of CREB, which binds to the cAMP response element (CRE) within the MITF promoter region, thereby increasing *MITF* expression [35]. Additionally, the PKC pathway can activate the MAPK pathway by activating Raf [36], leading to an increase in *MITF* expression.

### NO/cGMP signaling pathway

**Figure 2.**
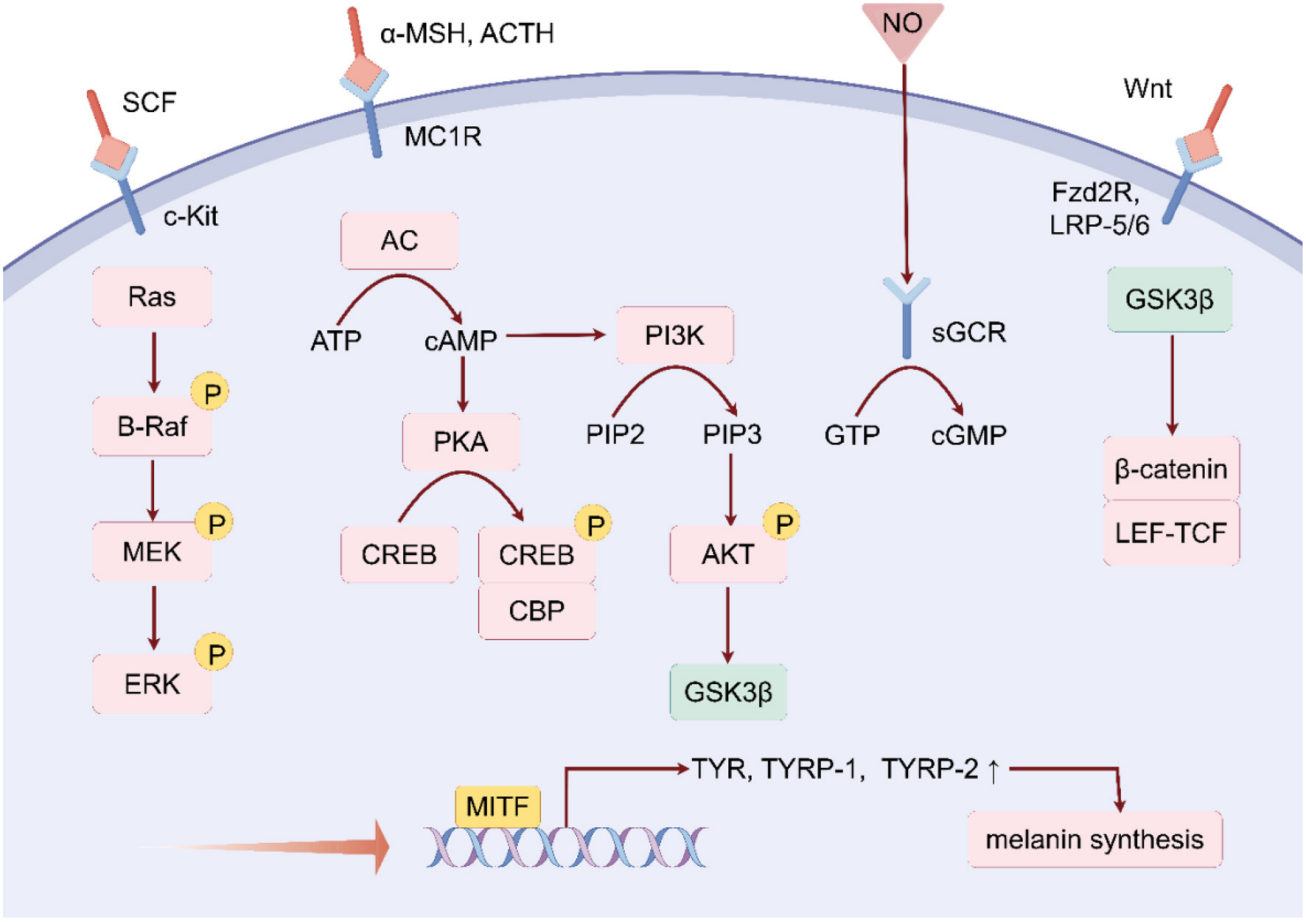
**Signaling pathways regulating melanin synthesis.** The regulation of melanin synthesis is associated with various signaling pathways, including the MC1R/α-MSH signaling pathway, the PI3K/Akt signaling pathway, the Wnt/β-catenin signaling pathway, the MAPK signaling pathway, and the NO signaling pathway. SCF: Stem cell factor; MC1R: Melanocortin 1 receptor; α-MSH: α-Melanocyte stimulating hormone; ACTH: Adrenocorticotropic hormone; NO: Nitric oxide; GSK3β: Glycogen synthase kinase 3 β; sGCR: Soluble guanylate cyclase receptors; cGMP: Cyclic guanosine monophosphate; PKA: Protein kinase A; AC: Adenylyl cyclase; PI3K: Phosphoinositide 3-kinase; CBP: CREB-binding protein; MITF: Microphthalmia-associated transcription factor; TYR: Tyrosinase; TYRP-1: Tyrosinase-related protein 1; TYRP-2: Tyrosinase-related protein 2; LRP-5/6: Lipoprotein receptor-related protein 5/6; LEF-TCF: Lymphoid enhancer factor/T-cell factor.

NO is a diffusible free radical that acts as an autocrine and paracrine molecule that regulates cellular functions [37, 38]. NO binds to soluble guanylate cyclase receptors (sGCR) produced by keratinocytes, enhancing their activity and catalyzing the conversion of GTP into the intracellular second messenger cyclic guanosine monophosphate (cGMP). cGMP can induce *MITF* expression and melanin production [39] and activate protein kinase G (PKG), thereby enhancing *MITF* expression and regulating melanin synthesis [40] (Figure 2).

## Overview of CLD

The development of CLD is linked to several factors, the most prevalent of which are alcohol abuse, obesity or metabolic disorders, autoimmune hepatitis, and viral hepatitis (specifically HBV and HCV) [1]. CLD encompasses viral hepatitis, alcoholic liver disease (ALD), autoimmune hepatitis, NAFLD, NASH, and cholestatic liver diseases, among others [41–43].

Liver fibrosis, characterized by excessive deposition of the extracellular matrix (ECM) in the liver tissue, is a reparative response to liver parenchymal injury. It represents the final stage of progression in CLD and is a critical step toward cirrhosis [43].

## Mechanisms linking CLD to skin hyperpigmentation

### Oxidative stress in CLD

Many factors contribute to liver fibrosis, including oxidative stress and inflammatory cytokines (Table 1). In the course of CLD, the levels of these factors in the serum increase, and their elevation is associated with enhanced melanin synthesis, suggesting that they may be potential causes of the increased melanin synthesis observed during the progression of CLD.

**Table 1:** Functions of factors in CLD and skin pigmentation

**Factor**	**Relationship with liver fibrosis**	**Relationship with pigmentation**	**References**
ROS	Induces hepatocyte necrosis/apoptosis, activates hepatic stellate cell proliferation/migration/collagen accumulation, promotes liver fibrosis, stimulates inflammatory factor release, exacerbates liver fibrosis	Activates the Akt/NF-κB pathway and the Wnt/β-catenin signaling pathway	[45–48, 60, 61, 170]
RNS	Increases with the severity of liver cirrhosis	Activates the NO signaling pathway	[85, 86, 171, 172]
IL-1α	Leads to liver inflammation and the expression of hepatic inflammatory cytokines	Increases TYR content when acting with the existence of KGF	[94–96]
IL-10	Increases in patients with ACLF	Activates the PI3K/Akt pathway and the JAK/Stat3 pathway	[99, 100]
IL-18	Exacerbates pathological processes in liver fat tissue, induces collagen deposition in HSCs	Activates the p38/MAPK and PKA pathways	[103–105, 173, 174]
IL-33	Promotes fibrosis mediated by HSCs, increases in patients with liver fibrosis	Activates the MAPK and PKA pathways	[112–115]
PGs	Correlate with viral load/severity in chronic HBV, increase in NASH patients	Increase dendricity in melanocytes, enhance TYR activity	[117–121]
GM-CSF	Increases in patients with ACLF	Increases the proliferation of melanocytes and the synthesis of melanin	[8, 99]
SCF	Promotes liver regeneration, plays a role in liver fibrosis, increases in patients with CHC	Activate the MAPK/ERK and PI3K/AKT pathways,	[128–130, 175]
ET-1	Increases in patients with NAFLD, correlates positively with the degree of liver fibrosis	Reduces the generation of H_2_O_2_, activates the PKC pathway	[86, 133, 134, 176]
SHBG	Decreases in postmenopausal women with NAFLD, enhances hepatic steatosis	Decreases intracellular cAMP levels in melanocytes, inhibits the activity of TYR, activates the PI3K/AKT pathway	[137–140]
Estrogen	In patients with chronic liver disease, liver cell function declines, leading to reduced deactivation of estrogen	Increases TYR activity	[152, 153, 177, 178]
Gln	Decreases in NAFLD patients, increase in in acute liver failure patients	Reduces ROS, activate the Nrf2/ARE pathway. Decreases the dephosphorylation of the Ser-473 site on AKT, inhibits the PI3K/AKT pathway	[158–160, 163, 179, 180]

CLD: Chronic liver disease; ROS: Reactive oxygen species; NO: Nitric oxide; RNS: Reactive nitrogen species; IL-1α: Interleukin-1α; TYR: Tyrosinase; IL-10: Interleukin-10; IL-18: Interleukin-18; IL-33: Interleukin-33; ACLF: Acute-on-chronic liver failure; PI3K: Phosphoinositide 3-kinase; MAPK: Mitogen-activated protein kinase; PKA: Protein kinase A; HSCs: Hepatic stellate cells; PGs: Prostaglandins; NASH: Non-alcoholic steatohepatitis; GM-CSF: Granulocyte-macrophage colony-stimulating factor; SCF: Stem cell factor; CHC: Chronic hepatitis C; NAFLD: Non-alcoholic fatty liver disease; SHBG: Sex hormone-binding globulin; Gln: Glutamine; NrF2: Nuclear factor E2-related factor-2.

### Role of reactive oxygen species (ROS)

Under normal physiological conditions, the antioxidant system maintains a balance with oxidative processes to prevent cell damage. In pathological states, oxidative stress products react with DNA, lipids, and proteins, leading to cell death. Oxidative stress is a critical step in liver fibrosis [44]. Excessive ROS induced by oxidative stress can cause hepatocyte necrosis or apoptosis and activate proliferation, migration, and collagen accumulation in hepatic stellate cells (HSCs) [45], resulting in liver dysfunction and excessive ECM deposition, leading to diffuse liver fibrosis [46].

Hepatocyte necrosis can also release inflammatory cytokines, such as tumor necrosis factor α (TNF-α) and transforming growth factor β (TGF-β) [47]. Excessive ROS can further aggravate liver fibrosis by activating NF-κB, promoting the release of inflammatory cytokines, such as TNF-α and interleukin-6 (IL-6) [48]. Concurrently, a series of antioxidant systems are activated, and excessive ROS can activate the nuclear factor E2-related factor-2/heme oxygenase-1 (Nrf2/HO-1) signaling pathway, exerting antioxidant effects and alleviating liver fibrosis [49].

Steps involved in oxidative reactions during melanin formation include tyrosinase-catalyzed oxidation of tyrosine to dopa, generating O^2-^, and tyrosinase-catalyzed oxidation of DHI to produce H_2_O_2_ [50]. Current studies have focused on controlling oxidative stress to improve CLD. Although the effects vary across different types of CLD, they remain a potential strategy for managing disease progression [51].

Studies on liver diseases such as melasma [52, 53] and post-inflammatory hyperpigmentation (PIH) [54] and their relationship with oxidative stress have been suggested and awaits further confirmation through research. This indicates a connection between oxidative stress generated during CLD and melanin deposition. Once oxidative stress is activated, a cascade of antioxidant systems is subsequently triggered. Increased melanin synthesis may occur as a result of the activation of signaling pathways within the antioxidant process, leading to excessive pigmentation [55]. Low concentrations of H_2_O_2_ have been shown in studies to promote melanin synthesis and its transfer to keratinocytes [56, 57].

Under oxidative stress, such as ultraviolet light and other stimuli, p53 is activated in keratinocytes, leading to the synthesis of higher levels of SCF, ET-1, POMC, and α-MSH, which promote melanin production. Additionally, fibroblasts synthesize and secrete more NGF-β and neuregulin-1 to regulate melanin production through a paracrine mechanism [58].

Growing evidence indicates that at low concentrations, ROS serve as secondary messengers in diverse cellular processes and facilitate redox-dependent events [59]. Under the influence of ROS, multiple signaling pathways related to melanin synthesis may be activated, including the Wnt/β-catenin, PI3K/Akt, ERK1/2, and Nrf2-ARE pathways [60–63]. Research has indicated that hypoxia induces the migration of HSCs and portal vein myofibroblasts (MFs). This process involves early activation of the ERK signaling pathway, which is mediated by mitochondria-dependent ROS. Additionally, immunohistochemical analyses of liver tissue in HCV-related fibrosis and cirrhosis suggest that MFs may be exposed to hypoxic conditions and oxidative stress *in vivo* [64]. However, there is very limited evidence regarding the relationship between oxidative stress and melanin deposition in CLD. Further studies are needed to determine whether oxidative stress conditions in CLD patients increase melanin deposition through activation of the aforementioned pathways.

It is worth noting that oxidative stress can activate the Nrf2 signaling pathway to mitigate oxidative stress, but this pathway has a negative regulatory effect on melanin synthesis. Nrf2 is constantly expressed in all skin cell types and its activity is regulated by ubiquitination and proteasomal degradation mediated by Kelch-like ECH-associated protein 1 (KEAP1) [65]. The reactive cysteines in KEAP1 function as redox sensors, and modifications by ROS and electrophiles induce conformational changes that promote the release, stabilization, and nuclear translocation of Nrf2 [66]. In diseases characterized by hypopigmentation, such as vitiligo, this pathway is considered a regulatory mechanism underlying abnormal pigmentation [67]. In patients with vitiligo, polymorphisms in the *Nrf2* promoter are associated with an increased risk of disease onset, and upregulation of *Nrf2* gene expression can be observed [68–70]. Keratinocytes and melanocytes upregulate Nrf2 expression in response to oxidative stress and other damaging environments, thereby activating the Nrf2-ARE pathway to alleviate oxidative stress. Studies have shown that Nrf2 can reduce melanin synthesis by inhibiting TYRP-1, suggesting that Nrf2 activators could potentially mitigate excessive pigmentation [71–73]. Additionally, excessive ROS levels can cause melanocyte death, leading to pigment loss [74].

Therefore, it can be speculated that the concentration and rate of increase of ROS may affect the regulation of melanin synthesis, leading to the dual roles of ROS in melanin regulation. For example, prolonged low-intensity oxidative stress may enhance melanin deposition, whereas short bursts of high-intensity oxidative stress may reduce melanin synthesis. However, the specific conditions require further research for confirmation.

Autophagy contributes to the maintenance of redox balance by recycling damaged macromolecules and organelles during oxidative stress, aiding cellular adaptation, and reducing oxidative damage. This process is predominantly mediated by the Nrf2 pathway, where Nrf2 degradation is inhibited by oxidative stress, thereby activating the Nrf2-ARE pathway [75, 76].

### Reactive nitrogen species (RNS)

RNS are formed by the interaction of NO with other compounds, including ROS, resulting in a series of highly oxidizing radicals and nitro compounds. NO is generated by nitric oxide synthase (NOS), which catalyzes the conversion of L-arginine to oxygen [77]. As a gas, NO can enter cells via diffusion or bind to cell surface receptors to modulate cellular functions. Another form of NO exists as a nitric oxide radical (NO), which transforms into NO through a series of electron transfer processes, ultimately leading to protein nitrosylation and affecting protein function.

NAFLD can be classified into two types: non-alcoholic fatty liver (NAFL), which is characterized by the absence of inflammation, and NASH, which is characterized by the presence of inflammation. Patients with NASH often experience hepatocellular injury and are more prone to liver fibrosis, which worsens with disease progression [78].

Research indicates that patients with NAFLD and NASH have higher levels of NO in serum than non-NASH patients [79]. Additionally, other studies have shown that exhaled nitric oxide (eNO) levels are elevated in patients with cirrhosis, and eNO levels increase with the severity of cirrhosis. This phenomenon may be related to endotoxin and cytokine stimulation of NOS to produce NO [80, 81]. NO is a major source of NO, and its generation is a prerequisite for protein nitrosylation. NO can directly nitrosylate tyrosine residues through a two-electron transfer following replacement of hydrogen at the third position of the phenyl ring, ultimately forming 3-nitrotyrosine (3-NT) [82]. Studies have found elevated levels of 3-NT in models of liver fibrosis induced by various chronic stimuli, including carbon tetrachloride and fructose [83, 84]. Similarly, the levels of 3-NT in the liver tissue of patients with chronic cirrhosis are also elevated [85], suggesting the occurrence of protein nitrosylation.

**Figure 3.**
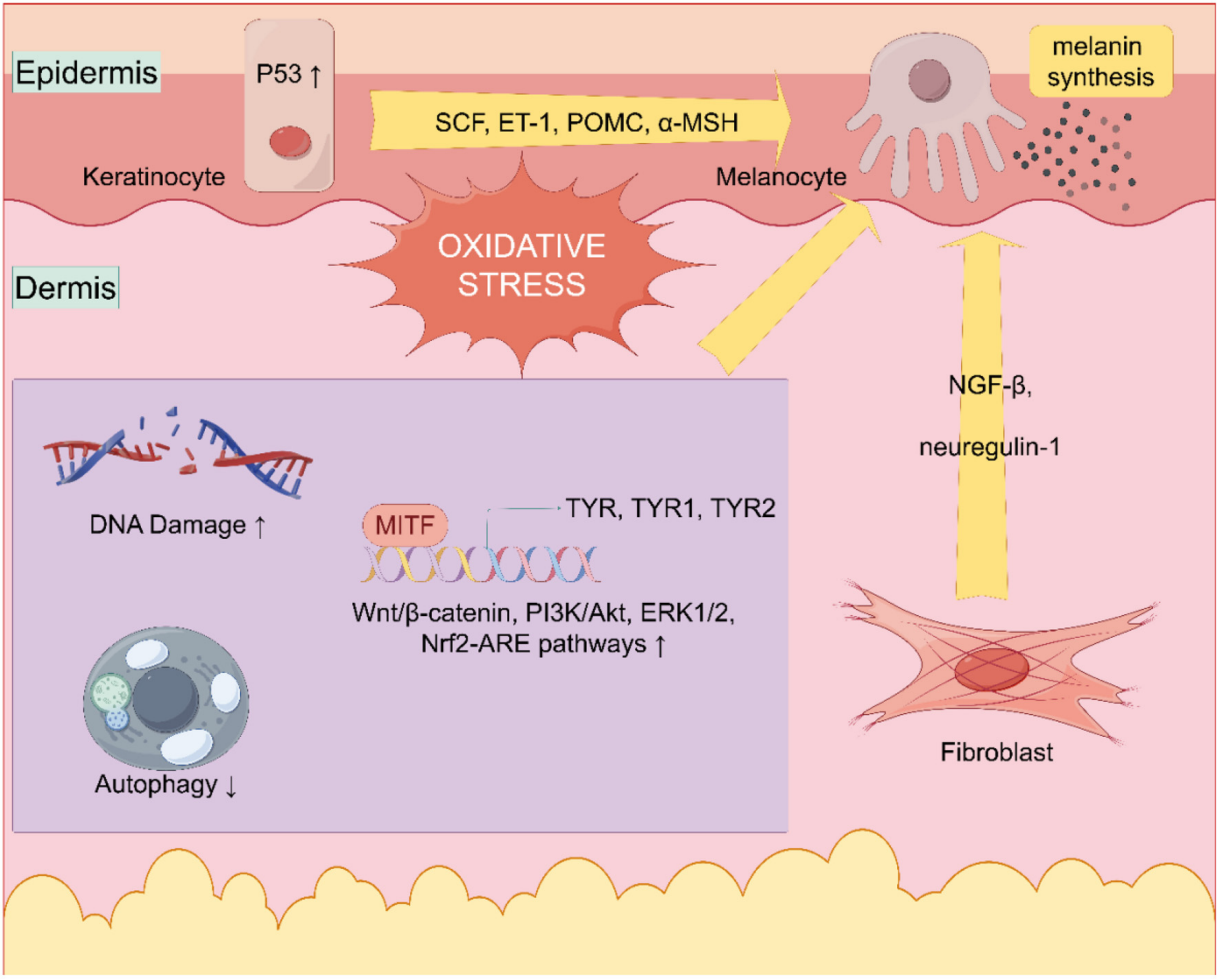
**Role of oxidative stress in hyperpigmentation disorders.** Under oxidative stress, various cells and inflammatory factors are involved in the response, primarily keratinocytes, melanocytes, and fibroblasts. SCF: Stem cell factor; α-MSH: α-Melanocyte stimulating hormone; ERK1/2: Extracellular signal-regulated kinase 1/2; MITF: Microphthalmia-associated transcription factor; PI3K: Phosphoinositide 3-kinase; NrF2: Nuclear factor E2-related factor-2; TYR: Tyrosinase; TYRP-1: Tyrosinase-related protein 1; TYRP-2: Tyrosinase-related protein 2.

NO is also involved in regulating melanin synthesis. NO in the cytoplasm of melanocytes can elevate *MITF* expression through cGMP, leading to increased expression of TYR, TRP-1, and TRP-2, thereby enhancing melanin synthesis [86] (Figure 3).

### Inflammatory cytokines and melanogenesis

PIH is a common occurrence; however, its exact cause is not fully understood. Research has identified that various inflammatory factors can promote melanin synthesis [86, 87], and some of these factors tend to increase during chronic inflammation. This suggests that the release of inflammatory factors in CLD may be associated with pigmentary changes.

Chronic low-grade inflammation mediated by inflammatory factors plays a crucial role in the pathophysiology of the disease. Imbalance between elevated pro-inflammatory cytokine levels and reduced anti-inflammatory cytokine levels is a pivotal step in the progression from simple steatosis to steatohepatitis, advanced liver fibrosis, and cirrhosis [88, 89].

Furthermore, studies have indicated that inflammatory factors can directly or indirectly influence skin pigmentation, with both promoting and inhibiting effects [90, 91]. Various skin cells, such as keratinocytes and fibroblasts regulate melanin synthesis through paracrine actions. Interleukin-18 (IL-18), Interleukin-33 (IL-33), granulocyte-macrophage colony-stimulating factor (GM-CSF), prostaglandin E2 (PGE2), and prostaglandin F2 (PGF2) secreted by keratinocytes and fibroblasts promote melanin synthesis [92, 93]. Multiple inflammatory factors modulate skin pigmentation via various signaling pathways.

However, there is a lack of evidence to support a direct correlation between increased levels of inflammatory factors and melanin deposition in CLD, and further experimental validation is needed.

### Interleukin-1**α** (IL-1**α**)

In experiments with hypercholesterolemic mice, it was found that the absence of *IL-1α* gene expression in Kupffer cells alleviated liver inflammation, suggesting a role for IL-1α secretion from Kupffer cells in NAFLD [94]. Additionally, studies have indicated that IL-1 is a major factor contributing to liver inflammation and expression of hepatic inflammatory cytokines [95].

IL-1 stimulates fibroblasts to produce KGF, which increases *TYR* expression in melanocytes. In this experiment, human full-thickness skin grafts and healthy human facial skin transplanted into SCID mice were cultured with additional IL-1, KGF, or a combination of both. These results showed that IL-1 alone had no effect on melanin synthesis. However, the experimental group with KGF and the group treated solely with KGF exhibited increased melanin synthesis, with the combination treatment group showing the most significant increase [96].

However, in melanoma cell lines LB2259-MEL and CP50-MEL treated with IL-1β, IL-1β might reduce the expression of *MITF* through the NF-κB and JNK pathways, and this reduction is associated with the expression of the *IL-1* receptor type I (*IL-1RI*) gene in melanoma cells [97, 98].

Therefore, further research is required to determine whether IL-1 promotes melanogenesis in CLD, which may depend on the presence of KGF during disease progression. In addition, the mechanism by which KGF interacts with IL-1 to promote melanin synthesis requires further investigation.

### Interleukin-10 (IL-10)

During chronic progression of liver disease, infections or gastrointestinal bleeding can trigger a reversible decompensated state known as ACLF. Excessive pigment deposition also occurs in ACLF as an aberrant condition of liver disease, aiding in early disease diagnosis through the identification of abnormal pigmentation [4–6]. Elevated levels of IL-10 can be measured in the serum of ACLF patients [99].

In the regulation of melanin synthesis, IL-10 can activate the PI3K/Akt pathway and the JAK/Stat3 pathway. The activation of the PI3K/Akt pathway subsequently activates the classical NF-κB pathway and deactivates GSK-3β. Consequently, this process further upregulates melanin production [100].

Therefore, the increase in IL-10 in the serum of ACLF patients may enhance melanin synthesis through the PI3K/Akt signaling pathway. Moreover, the clinical manifestation of melanin deposition holds promise as a potential predictor of ACLF. Further research is necessary to confirm the direct relationship between these factors.

### Interleukin 18 (IL-18)

IL-18, a member of the IL-1 family, is secreted by various inflammatory cells, such as neutrophils, T lymphocytes, and B lymphocytes, and functions as a chemotactic factor [101].

In patients with cirrhosis, serum IL-18 levels are significantly elevated compared with those in healthy controls [102]. Experimental studies in IL-18 knockout mice have demonstrated the development of NAFLD, which can be ameliorated by recombinant IL-18, indicating its role of IL-18 in controlling the onset and progression of NAFLD [103]. When IL-18 signaling is blocked by its natural antagonist, IL-18BP, the activation of HSC triggered by NLRP3 inflammasome activation is eliminated. Thus, IL-18 directly influences HSC activation and may serve as a target for IL-18-based therapies for liver fibrosis [102].

IL-18 has also been shown to promote melanin deposition. IL-18 activates the p38/MAPK and PKA pathways to increase expression of *MITF*, *TYRP-1*, and *TYRP-2*, thereby enhancing melanin synthesis [104, 105].

In summary, IL-18 can be considered a potential therapeutic target for NAFLD, as it can enhance melanin synthesis through the p38/MAPK and PKA pathways. However, the direct role of IL-18 in promoting melanin synthesis in CLD requires further investigation.

### Interleukin 33 (IL-33)

IL-33, also a cytokine of the IL-1 family, primarily maintains cellular homeostasis and mediates adaptive immune responses [106]. The receptor for IL-33, ST2, is expressed on the surface of epithelial cells, HSCs, endothelial cells, and other cell types, with studies showing richer expression in keratinocytes and fibroblasts [107, 108]. When IL-33 acts on mast cells, it induces the production of various cytokines and activates immune cells, such as macrophages, leading to inflammation [109, 110].

During the acute phase of liver disease, IL-33 exhibits a protective effect on hepatic cells, helping maintain cellular homeostasis [111]. However, during the progression of CLD, IL-33 promotes fibrosis mediated by HSCs through a series of cascading reactions [112]. Studies have confirmed significantly elevated levels of IL-33 and ST2 mRNA in the serum of patients and mice with liver fibrosis compared to those in controls [113, 114].

Regarding melanin deposition, IL-33 can activate the MAPK and PKA pathways to increase the expression of *MITF*, *TYRP-1*, and *TYRP-2*, thereby promoting melanin deposition in the skin [115].

Therefore, the role of IL-33 in promoting liver fibrosis and its elevation in CLD, along with its stimulatory effect on melanin synthesis, is supported by research. However, the evidence regarding its direct involvement in CLD and melanin deposition remains insufficient and requires further exploration.

### Prostaglandins (PGs)

PGE2 is metabolized from the ω-6 polyunsaturated fatty acid arachidonic acid, with the liver serving as the main organ for PGE2 metabolism and expressing receptors to maintain hepatic homeostasis [116].

HBV infection is a major cause of CLD, leading to liver fibrosis and HCC in some cases. In patients with chronic HBV-infected infection, serum concentrations of PGE2 correlate positively with viral load and liver damage severity [117]. In NAFLD, serum levels of PGE2 increase in patients with NASH [118].

Experimental studies have shown that both PGE2 and prostaglandin F2α (PGF2α) can increase dendrites in melanocytes, and PGF2α can enhance TYR activity, thereby promoting skin pigmentation [119, 120]. It has been demonstrated that under the influence of PGE2, dendrites of melanocytes increase and the granule content in shed vesicles increases, although PGE2 has a minimal effect on keratinocytes [121].

Thus, the serum levels of PGE2 are elevated in certain liver diseases and can enhance melanin synthesis by promoting the proliferation of dendritic processes in melanocytes and increasing the activity. These findings suggest that PGE2 primarily affects skin pigmentation through its actions on melanocytes, indicating its potential role in melanin deposition in CLD.

### Granulocyte-macrophage colony-stimulating factor (GM-CSF)

In liver diseases, such as ACLF, the quantity, phenotype, gene expression, and function of neutrophils correlate with patient prognosis, with higher neutrophil counts associated with increased short-term mortality risk and reduced phagocytic function [122–124]. Studies have shown elevated circulating neutrophil counts and percentages in patients with ACLF compared to controls, accompanied by significantly increased plasma concentrations of GM-CSF [99].

Under UV-B stimulation, keratinocytes secrete GM-CSF via paracrine signaling, which promotes the proliferation and synthesis of melanocytes [8]. Therefore, the stimulatory effect of GM-CSF on melanin synthesis under UV-B stimulation suggests a potential association with the development of “liver facies” in CLD. Further research is required to confirm this conclusion.

### Other factors influencing melanogenesis in CLD

#### Stem cell factor (SCF)

SCF, also known as c-Kit ligand, is a hematopoietic growth factor involved in hematopoiesis, gametogenesis, and migration of melanocytes from the neural crest to the epidermis [125]. SCF also promotes the synthesis of proinflammatory cytokines, chemokines, and histamine in mast cells [126].

During CLD, SCF collaborates with GM-CSF to promote liver regeneration and plays a role in liver fibrosis [127]. Research indicates that serum SCF levels are significantly elevated in patients with chronic hepatitis C (CHC) compared to controls, suggesting SCF’s involvement of SCF in liver repair processes in patients with CHC [128].

Binding of SCF to c-Kit activates several signaling pathways, including MAPK/ERK, PI3K/AKT, and JAK/STAT [129]. Activation of Ras/MAPK leads to MITF phosphorylation, which increases the expression of enzymes involved in melanin synthesis and promotes melanosome transport, thereby enhancing melanogenesis [130].

In conclusion, SCF and GM-CSF are involved in liver regeneration and repair, with elevated levels found in the serum of patients with CHC. Additionally, they can activate MAPK/ERK and PI3K/AKT pathways, leading to increased melanin synthesis. This suggests that they may play a promoting role in the development of “liver facies.”

#### Endothelin 1 (ET-1)

Endothelin (ET), originally extracted from endothelial cells, is a vasoconstrictive peptide with functions, including vascular contraction and promotion of cell proliferation, serving as a crucial regulator of cardiovascular function [131, 132]. Clinical studies have indicated significantly elevated serum levels of ET-1 in patients with NAFLD compared to controls, with the magnitude of increase correlating positively with the degree of liver fibrosis [133].

In the regulation of melanogenesis, ET-1 activates the PKC pathway upon binding with ETR, promoting the expression of *TYR*, *TRP-1*, and *TRP-2* [86]. *In vitro* experiments demonstrate increased *MITF* expression and significantly enhanced melanin synthesis in cells cultured with ET-1 [134, 135]. This suggests that elevated serum ET-1 levels in CLD may be one of the factors contributing to increased melanin synthesis.

#### Sex hormone-binding globulin (SHBG)

SHBG is a glycoprotein produced by the liver with high affinity for testosterone and plays a role in the regulation of androgens [136]. Research on postmenopausal women with NAFLD has shown lower serum SHBG levels than those in control groups [137]. Additionally, in men, hepatic steatosis is associated with decreased testosterone levels, which in turn correlates with reduced SHBG levels [138]. Thus, the interaction between SHBG and androgens plays a role in NAFLD pathogenesis and may be mutually influential.

Experimental findings indicate that in the presence of SHBG and androgens, such as testosterone, 5α-dihydrotestosterone, and methyltrienolone (R1881), cAMP levels decrease in the melanocytes of normal individuals. Binding of testosterone and R1881 to SHBG mildly inhibited TYR activity [139]. Furthermore, in insulin-resistant cells, *SHBG* expression decreases when the PI3K/AKT pathway is activated [140].

Therefore, reduced SHBG expression during NAFLD may lead to increased melanin synthesis through the activation of the PI3K/AKT pathway and enhanced TYR activity. Further experimental evidence is needed to determine whether SHBG is associated with melanin deposition in CLD.

#### Estrogen

Estrogens can be classified into three types: 17β-estradiol (E2), which is synthesized from cholesterol in the ovaries of premenopausal women, and the metabolites estrone (E1) and estriol (E3), which are derived from the metabolism of E2. Among these, E2 exhibits the highest activity [141]. Estrogen receptors (ER) are expressed not only in organs, such as the ovaries, breasts, and uterus, but also in the liver, primarily as ER-α [142].

Estrogen is primarily metabolized in the liver. Specific manifestations of CLD, such as the development of spider nevi and abnormal enlargement of the male breast, result from reduced estrogen inactivation owing to impaired liver function [143, 144]. In patients with cirrhosis, plasma estrogen levels are higher than those in control groups [145, 146].

Similar to CLD, abnormal pigmentation is also observed in pregnant women and is suspected to be caused by increased estrogen levels [147, 148]. Moreover, ER-β expression is increased in the lesional areas of patients with lentigines, indicating that estrogen is involved in melanin enhancement [149]. This suggests that melanin deposition in patients with CLD may be related to a reduction in estrogen inactivation. Estrogen can regulate melanin synthesis by acting on ER, leading to an increase in melanin production. Additionally, G protein-coupled ER may also be involved in the regulation of melanin by estrogen, exerting effects independent of the ER [150]. In mouse B16 melanoma cells, estradiol increased cell proliferation, melanin synthesis, tyrosinase activity, and expression of the tyrosinase family and *MITF*. This effect is associated with the activation of the cAMP-PKA pathway and the upregulation of the expression and activity of the melanin-synthesizing enzymes tyrosinase and *MITF* [151]. *In vitro* experiments have shown that cells exposed to E2 exhibit increased TYR activity. This increase in TYR activity correlates with enhanced melanin synthesis [152]. Melanocytes treated with E2 showed a dose-dependent increase in viability after 24 h of incubation. The maximum response varied between 145% and 213% of the baseline activity depending on the source of the donor cells [153].

Therefore, it is suggested that, during the progression of CLD, reduced estrogen metabolism may lead to interactions with melanocytes, resulting in increased TYR activity and subsequent melanin synthesis, leading to pigment deposition.

#### Glutamine (Gln)

Hepatic encephalopathy plays a critical role in the late stages of CLD, and its occurrence and progression are closely related to the increase in blood ammonia levels resulting from liver dysfunction. The body’s ammonia detoxification pathways include the urea cycle in the liver and Gln synthesis system, primarily in the skeletal muscle and brain. The liver converts toxic ammonia into urea via the urea cycle [154]. During the progression of CLD, liver metabolic function is impaired, and early on, the Gln synthesis system in the skeletal muscle and other tissues compensates, preventing an increase in blood ammonia levels until the decompensation stage, when blood ammonia levels rise [155]. Before changes in blood ammonia levels occur, amino acid metabolism disorders and liver fibrosis have already appeared [156]. Increased Gln metabolism breakdown leads to increased ammonia production and further requires urea synthesis, exacerbating the liver burden and accelerating liver disease progression [157].

Metabolic changes in Gln levels may differ between the early stages of liver disease and acute liver failure. Studies have shown that in patients with NAFLD, decreased liver Gln synthetase is associated with disease severity, and elevated glutamate/Gln ratios in the liver and blood indicate increased Gln breakdown [158, 159]. However, in patients with acute liver failure, increased net uptake of muscle ammonia and an increase in Gln net production have been observed [160]. Therefore, in the early stages of liver disease, a decrease in Gln levels can be inferred, whereas in the acute liver failure phase, an increase in Gln levels is suggested.

Sufficient Gln maintains normal GSH levels, thus buffering oxidative damage. Additionally, during Gln metabolism, NADPH is provided to maintain GSH in its reduced state, thus assisting in its antioxidant action [161, 162]. Phosphorylation of the Ser-473 site of AKT is a prerequisite for its complete activation [163]. ROS can cause dephosphorylation of the Ser-473 site on AKT, thereby inhibiting the PI3K/AKT pathway [164, 165].

In patients with acute liver failure, increased Gln production leads to reduced ROS levels. Consequently, diminished inhibition of the PI3K/AKT pathway by ROS may contribute to increased melanin deposition in patients with acute liver failure.

However, the reduction in Gln during the early stages of CLD may lead to a decrease in melanin synthesis. Research has found that Gln can reduce ROS production in melanocyte stress models and activate the Nrf2/ARE pathway, which negatively regulates melanin synthesis [67]. Therefore, in the early stages of CLD, Gln may not be the primary factor that regulates the increase in melanin synthesis.

In summary, an increase in Gln was positively correlated with melanin synthesis. Thus, it can be inferred that elevated Gln levels during the acute liver failure stage may be associated with hyperpigmentation. Further research is needed to confirm the levels of Gln and its impact on melanin deposition across the different stages of liver disease.

## Clinical implications

### Early detection of CLD through skin pigmentation

Skin pigmentation changes have emerged as notable clinical manifestations associated with ACLF and CLD progression [4]. This suggests that hyperpigmentation may serve as an important indicator of liver disease deterioration and onset of acute liver failure, highlighting its potential diagnostic value for both conditions. Therefore, when the clinical manifestations of pigmentation are combined with the elevation of related factors to form a scoring system, it could enhance the prediction of the stages of CLD progression, thereby improving the accuracy and specificity of these predictions. This integrated approach may provide valuable insights into the early detection and noninvasive monitoring of liver disease, potentially leading to timely interventions.

However, despite the identification of various factors that are elevated in CLD, there is still a lack of experimental and clinical data confirming whether these elevations positively correlate with the severity of liver disease progression. Furthermore, it remains unclear whether these factors show significant increases during acute liver failure, and how they relate to the observed increase in skin pigmentation.

Consequently, there is a pressing need for more clinical reports to investigate the sequence of liver disease manifestations and their correlation with disease deterioration, as well as to establish a direct relationship between the factors discussed and increased skin pigmentation in CLD. Such research could validate skin pigmentation changes as a valuable tool for early detection of CLD.

### Therapeutic considerations

Current studies have focused on controlling oxidative stress to improve CLD. For instance, silymarin and vitamin E provide a certain degree of protection against oxidative stress injury in CLD [166, 167]. Additionally, substances, such as Veronica ciliata Fisch, Pu-erh tea extracts, luteolin-7-O-glucoside, quercetin, and gallic acid have been shown to provide a certain degree of protection against oxidative stress in CLD [51]. However, whether the suppression of oxidative stress in CLD treatment contributes to improvements in skin hyperpigmentation remains to be investigated. There is also growing interest in exploring whether similar therapeutic agents could effectively reduce pigmentation in the general population.

Similarly, many medications improve liver function by targeting specific signaling pathways or preventing the release of inflammatory factors. For example, pirfenidone (PF) has been shown to exert dose-dependent inhibition of the Wnt/β-catenin signaling pathway, thereby improving cholestatic liver injury [168]. Salvianolic acid B can inhibit inflammatory markers, including IL-1β, IL-6, TGF-β, TNF-α, and COX-2, and it can suppress the MAPK inflammatory signaling pathway, thereby alleviating cholestatic liver injury both *in vivo* and *in vitro* [169]. Additionally, some drugs inhibit melanin synthesis by targeting the signaling pathways described in this article. For example, defects in the Wnt/β-catenin signaling pathway exacerbate ferroptosis in melanoma by regulating MITF. Targeting the Wnt/β-catenin-MITF pathway may be a promising strategy for enhancing ferroptosis and improving the efficacy of anti-PD-1 immunotherapy [34]. Therefore, there is hope that research will yield drugs capable of delaying liver function deterioration, while also reducing skin hyperpigmentation.

Therefore, the potential effects of antioxidant and anti-inflammatory drugs, along with the other factors discussed in this article, on skin whitening require further investigation. Specifically, it is crucial to determine whether enhancing liver function can lead to a reduction in the serum levels of these factors, thereby facilitating a decrease in hyperpigmentation. Such studies could provide valuable insights into therapeutic approaches for managing skin pigmentation changes associated with liver diseases.

### Future research directions

There is an urgent need for clinical studies to validate the proposed mechanisms linking changes in skin pigmentation with CLD progression and acute liver failure. Specifically, further research is necessary to establish the correlation between elevated biomarkers and the severity of liver disease as well as to clarify how these factors contribute to increased skin pigmentation.

Additionally, investigating potential treatments targeting both liver dysfunction and skin pigmentation is essential. Therapeutic agents that control oxidative stress and inflammatory responses, such as silymarin, vitamin E, and PF, should be explored to improve the liver function and reduce hyperpigmentation. Understanding the relationship between enhanced liver function and serum levels of inflammatory markers may pave the way for new interventions that not only improve liver health but also address aesthetic concerns related to skin pigmentation changes in patients with CLD. Such integrative research could lead to innovative therapeutic strategies and improved patient outcomes.

## Conclusion

Skin hyperpigmentation is a notable clinical manifestation of CLD and ACLF; however, research on its pathogenesis remains limited. This article outlines the processes of melanin synthesis and transport, and provides examples of the signaling pathways activated during both CLD progression and melanin synthesis. It lists factors that are elevated in serum levels in CLD and are associated with increased melanin synthesis, specifically oxidative stress factors, inflammatory factors, and other factors. This suggests potential underlying reasons for the increased melanin synthesis observed in CLD. Understanding the sequence of liver disease manifestations and progression may yield important prognostic indicators for CLD. Future research should focus on the effects of antioxidant and anti-inflammatory treatments on skin whitening, particularly examining whether improvements in liver function can lead to decreased serum levels of the factors that contribute to hyperpigmentation. By elucidating these mechanisms, we can better identify excessive skin pigmentation as an early marker of disease progression, ultimately enhancing patient care and management of liver dysfunction.
